# The Post-Dictatorship Memory Politics in Portugal Which Erased Political Violence from the Collective Memory

**DOI:** 10.1007/s12124-018-9452-8

**Published:** 2018-07-28

**Authors:** Raquel da Silva, Ana Sofia Ferreira

**Affiliations:** 1 International development Department, University of Birmingham, Edgbaston, Birmingham, B15 2TT UK; 2 Instituto Universitário de Lisboa (ISCTE-IUL), Centro de Estudos Internacionais, Lisbon, Portugal; 3 Institute of Contemporary History, Universidade Nova de Lisboa, Lisbon, Portugal

**Keywords:** Memory, Political violence, Revisionism, Political narratives

## Abstract

Former clandestine militants’ voices and stories have been recurrently silenced in the Portuguese “battle over memory”, because their activities were linked to events, such as the Revolution of 25 April 1974, which have themselves been politically and socially depreciated in mainstream political narratives. Only recently did the traditional political narratives start to be questioned and debated by Portuguese scholars. Such political narratives took root in the country in the decades that followed the April Revolution, with various scholars and politicians denying the fascist categorisation of *Estado Novo* and adopting an authoritarian, non-totalitarian and non-fascist perspective, while recurrently depicting the Revolution as highly negative (namely as the source of the economic troubles of the country). Thus, for a long time, Portuguese conservatives opted to avoid debates on the 48 years of the *Estado Novo*’s regime which, among other things, maintained a very repressive and violent political police force, a camp of forced labour in Cape Vert known as *Tarrafal*, and a Colonial War on three African fronts. This article examines the existent academic publications which counter such oblivion of memory regarding armed struggle in Portugal. It also explores the reasons behind, on the one hand, the whitewashing of *Estado Novo* and the historical revisionism typical of the 1970s and 1980s, and, on the other hand, the “rebellion of memory” which emerged in the 1990s.

## Portuguese Armed Organisations in Context

The exercise of context reconstruction and exploration is greatly important for the understanding of how and why political violent organisations came into existence in Portugal (in different periods of time and in different political and social conditions), and how violence was legitimised by some and demonised by others.^1^

**Fig. 1:**
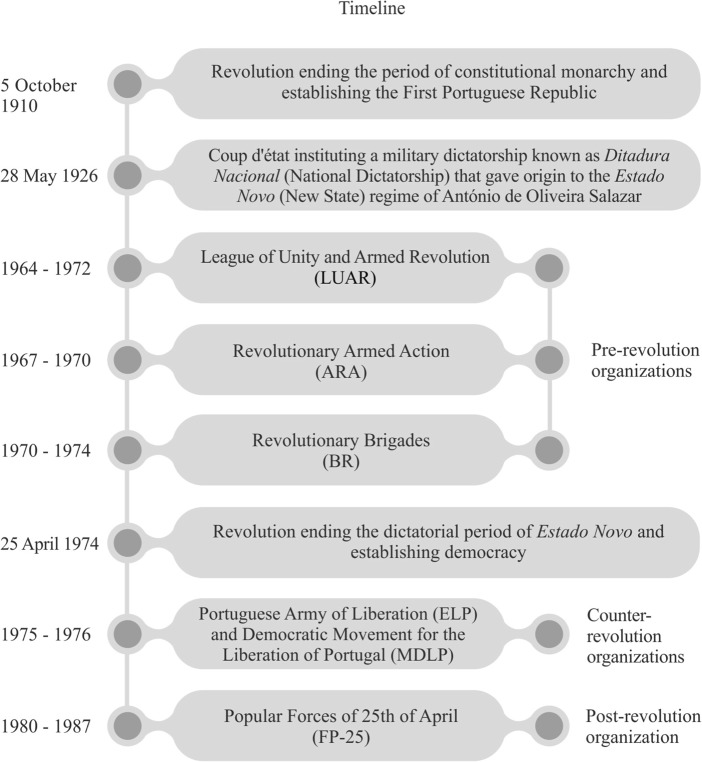
Timeline

The present article is solely focused on political violence committed by non-state actors and during three specific periods: pre-, counter- and post-revolution. During this time span of almost three decades (1962-1987), six different violent organisations emerged in Portugal. In the first wave (1962-1974), *Estado Novo,*^2^ in a very clear and direct way, determined the emergence of the LUAR (League of Unity and Armed Revolution), the ARA (Revolutionary Armed Action) and the BR (Revolutionary Brigades) – the three revolutionary organisations which fought against the regime and its policies, predominantly the ones related to imperial, colonial and capitalist standpoints. These organisations resorted to violence against a regime which in their eyes was extremely violent and repressive and which could not be defeated by pacifist means. The latter had been tried for years without success and at a high personal cost (e.g. arrests, torture, forced labour) (Antunes 1974; Narciso 2000; Mortágua 2013; Pimentel 2014). In the second wave (1975-1976), the rise of the reactionary organisations the ELP (Portuguese Liberation Army) and the MDLP (Democratic Movement for the Liberation of Portugal) was triggered by fear of a possible communist occupation following the Revolution and by disagreement with the decisions taken by the provisional government (e.g. decolonisation). These organisations were essentially composed of right-wing military personnel, who in the majority of cases leaned towards the deposed regime. In the third wave (1980-1987), the revolutionary organisation the FP-25 (Popular Forces of the 25th April) believed that the ideals defended by the Revolution were fading away, giving place to the return of the unjust capitalist society of the past. This was a situation that in their perspective only a socialist revolution could solve. The following figure (Fig. 1) provides a timeline that depicts both the main historical events which set the context for the rise of armed organisations in Portugal, and the period of action and characterization of these same organisations.

In addition, Table 1 sets the political context behind the activity of each armed organisation under analysis, as well as their main characteristics. It is important to recognise explicitly here that the literature available on the action of armed organisations in Portugal is very scarce, and does not allow an even exploration and presentation of information. This is due to the fact that material available about some organisations (e.g., details about the armed actions carried out) is not available about others.

**Table 1:** Portuguese armed organisations in context

Estado Novo (1926-1974)			
Organisation	ARA	LUAR	BR
Period of action^a^	1964-1972	1967-1970	1970-1974
Government	Estado Novo	Estado Novo	Estado Novo
Ideology	Armed arm of PCP	• Plural lefts • Anti-Salazarism	• Rupture with PCP • Anti-Stalinism • Marxism-Leninism • Dialectical materialism
Main goals	• Overthrow the regime • End colonialism	Overthrow the regime	• Overthrow the regime • End colonialism
Target(s)	• Political and military structures • No human lives	• Political and military structures • No human lives	• Political and military structures • No human lives
Type of actions	• Explosives	• Explosives • Bank robbery • Attempt to take control of a city	• Petards • Explosives • Leaflets distribution • Bank robbery • Support to the colonial war
Revolution (25th April 1974)			
Organisation	ELP	MDLP	FP-25
Period of action^a^	1975-1976	1975-1976	1980-1987
Government	Provisional Government	Provisional Government	• Coalition PSD, CDS, PPM (1980-1983) • Coalition PS and PSD (1983-1985) • PSD (1985-1995)
Ideology	• Connected to the Liberal Party • Anti-communism	• Connected to the Progress Party • Anti-communism	• Connected to the FUP • Marxist-Leninist • Small group of anarchists within the organization • Marxism-Leninism • Dialectical materialism
Main goals	• Stop communist invasion • Stop decolonisation	• Stop communist invasion • Decolonise differently (commonwealth type of thing)	• Stop capitalism • Fight injustice in the workplace • Attain socialism
Target(s)	• Communist headquarters • Communists	• Communist headquarters • Communists	• Business owners/managers • Representatives of ‘fascism’
Type of actions	• Explosives	• Explosives	• Explosives • Shooting at close range • Bank robbery

^a^According to the literature

### First Wave: Estado Novo

*Estado Novo* is a period of Portuguese history that has its origins in the military coup of 28 May 1926. This military coup ended the First Republic^3^ and established a military dictatorship in Portugal, which at first did not have a well-defined ideology, but did have a deep scepticism regarding the effectiveness of parliamentary democracy. The first months of the military dictatorship established which faction of the movement which deposed the First Republic would be in power – the liberal-republicans or the conservatives – a fight won by the conservatives on 9 July 1926 (Mattoso 1994). In this context, all attention and hopes turned to Salazar. He was an important figure from the Catholic Centre, who seemed to hold the key to the country’s financial problems^4^ and at the same time did not appear as a political threat, not exhibiting any political affiliation and even showing reticence in accepting a political position (Mattoso 1994). Thus, on 18 April 1928 Salazar assumed the Ministry of Finance for the second time.^5^ This made him a very powerful minister in the difficult financial conjuncture that Portugal was going through (Pinto 2010). Salazar did not, however, only have a financial solution for the country, which assigned balance to the its economy and kept him in the same position through several different governments (Mattoso 1994). He also had a political programme in mind – the foundation of a new political, economic and social order, based on an authoritarian state (Oliveira 1990).

In order to accomplish his mission, Salazar based the regime on “a mythical idea of nation and national interest” (Rosas 2001, p.1032). In addition, he aimed, similarly to fascist regimes in Europe, to create a new type of Portuguese people, regenerated by the regime’s ideology (Cabrera 2014) and a “new order”, which would end the liberal century and start the re-education of the Portuguese people in the context of a regenerated nation (Rosas 2001, p.1033).

In order to preserve such a “new order” and to exert its authority as wide as possible, *Estado Novo* fashioned and implemented different societal structures, which functioned as the “keepers of order”. Among those, three were central to trigger the activity of armed organisations in Portugal: 1) the absence of political freedom, represented by the existence of a single party – the National Union; 2) the absence of freedom of expression, represented by the censorship; and 3) the existence of a political police, responsible for the institutionalization of violence and for the forced labour camp Penal Colony of Cape Verde.

It is also important to highlight that in the popular discourse *Estado Novo* is often referred to as fascism. This label does not always receive support in academic circles because although it is considered to have been an authoritarian regime, *Estado Novo* did not portray all the characteristics of an ideal type of fascism.^6^ The same often happened to Spanish Francoism. This argument was countered by Enzo Collotti (1989), who considered that the restriction of the liberty of expression, the repression of the political opposition, the violence against civilians perpetrated by the political police, the corporativism that eradicated the labour movement’s autonomy, and the moralising nature of the state in its intent to control every soul are enough to classify this Portuguese regime as fascism.

The implementation of Salazar’s political intent resulted in a strong wave of opposition throughout the year of 1931, including revolts in Madeira, Azores and Guinea led by the *Reviralho,*^7^ student demonstrations against the dictatorship in Lisbon and Oporto, and the action of the liberal conservatives who still believed in constitutional normalisation through elections (Pimentel 2014). However, the Portuguese opposition during *Estado Novo* was always permeated with various difficulties, especially before the 1940s. These were years in which the regime was being consolidated, the repression strengthened and organised, the opposition dispersed and fascism expanded in Europe (Raby 1988). However, during World War II and its aftermath, the increasing international challenge to authoritarian regimes had consequences for the opposition in Portugal. In 1941, the Portuguese Communist Party (PCP) was able to set up a clandestine structure and a strategy of infiltration in the official trade unions. Across the 1940s and 1950s, different opposition movements emerged in Portugal, such as the Anti-Fascist National Unity Movement (MUNAF) and the Democratic Unity Movement (MUD). However, these organisations’ activities were completely restrained by the regime. In the beginning of the 1960’s, different groups of individuals exiled in South America (particularly in Venezuela and Brazil) instigated some very important actions against *Estado Novo*. Among these were the assault on the Portuguese ocean liner *Santa Maria* in 1961, the assault on the military quarter of Beja in 1962 and, in the same year, the diversion of the TAP aircraft responsible for the connection Casablanca-Lisbon, which flew at low altitude over Portugal, dropping leaflets denouncing the atrocities of the regime (Santos 2011).

The 1960s carried a new wave of worldwide uprisings that in Portugal coincided the beginning of the Colonial War in Africa in 1961, growth of internal instability with the replacement of Salazar by Marcelo Caetano in 1969, and increasing awareness of revolutionary movements around the world (Leitão and Pina 1975). These circumstances strongly influenced and intensified the political opposition in Portugal, exacerbating anti-fascist, anti-capitalist and anti-imperialist positions and originating a movement towards the release of people from an oppressive regime (Leitão and Pina 1975). At that time, the perception of armed struggle as a way out of an entrenched situation began to emerge as a possibility, gaining supporters and replacing the traditional reformist discourse^8^ (Bebiano 2006a). Thus, in this setting, three of the organisations under analysis in the present study – the LUAR, the ARA and the BR – took up arms and fought *Estado Novo* through political violent means.

### Second and Third Waves: Revolution and Democracy

The revolution of 25 April 1974 was the last leftist revolution in Europe, bringing an end to one of the lengthiest dictatorships of the twentieth century (Rezola 2007). At the micro level, such a revolution is often considered to be totally unexpected, as Narciso (2000), Santos (2011) and Mortágua (2013) describe in their memoirs. However, a macro analysis indicates many signs of a regime marked by deep crisis, particularly in its last decade, such as:

- The Roman Catholic church, one of the pillars of the regime, prompted different actions against the Colonial War,^9^ which were influenced by Pope Paul VI himself, who in 1970 received a group of liberation movements’ representatives from the Portuguese colonies, placing into question the validity of the regime’s positions (Bebiano 2006a);
- The young urban students and workers were increasingly politicized and influenced by extreme-left ideologies, in a context of industrialization, war and forced exile in foreign countries, and were targeted by a violent repression^10^ (Cruzeiro and Bebiano 2006; Loff 2006);
- The Colonial War on three different fronts, which started in 1961, in Angola, and spread to Mozambique and Guinea-Bissau, was draining the armed forces (Rezola 2007);
- The Portuguese in the colonies started protesting both against the regime and the armed forces which were not able to protect them from the violent attacks perpetrated by the liberation movements (Rezola 2007);
- The pressure of the international community on the regime was renewed, due to the denunciation of the Wiriyamu massacre in Mozambique by Adrian Hastings, a Roman Catholic priest (Hastings 1973);
- The armed forces attempted coup in March 1974 (Rezola 2007); and
- The economic difficulties Portugal was facing, particularly due to the war and to the oil crisis of 1973 (Rezola 2007).

Thus, Portugal could be compared to a “pressure cooker”, ready to explode by itself or with some encouragement (Pimentel 2011, p.22). Help that indeed came, but from an unexpected player. The player on which the regime had relied the most – the armed forces.

Thus, in 1973 the *Movement of Captains* was created. This resulted from discontent among the military in the face of the policies of Caetano’s government, namely the continuation of the Colonial War. In addition, February 1974 saw the launch of the book *Portugal and the Future* by General Spínola, who was at the time deputy chief of the General Staff of the Armed Forces. In the book, he stated that the only solution to the Colonial War was not armed, but political, through the recognition of the right of people to self-determination (Spínola 1974). This stance, taken by such an important figure, triggered an implosion in the regime. It caused the dismissal of the General, but it also realised the regime’s worst fear by leading the young captains to revolt.

Thus, from the moment of creation of the *Movement of Captains* in August 1973 onwards, a part of the military connected to General Spínola did not stop planning the military coup, which finally took place on 25 April 1974. This was a military coup that quickly became a social revolution amongst the Portuguese people, who flooded the streets of the country supporting the armed forces and claiming the end of the regime. These people were finally able to actively participate in the “transitional process towards a fully institutionalized democracy” which finished in April 1976 with the implementation of a new constitution (Loff 2014, p.3).

This revolution prompted deep structural transformations in Portugal, particularly at economic, social and cultural levels (Rezola 2007). However, it also triggered new waves of armed action in the country, initially starred by the ELP and the MDLP in 1975, and later, in 1980, by the FP-25. These are the three last organisations under analysis in the present study.

The waves of violence which occurred after the April Revolution were caused by the reaction of two different factions – the extreme right and the extreme left – to the changes introduced by the democratic process. The first wave took place in the summer of 1975 and was motivated by the belief (held by the conservative and pro-regime people in Portuguese society), that the April Revolution had opened up the possibility for the implementation of a “Marxist/Communist/collectivist/totalitarian dictatorship worse than Salazar’s” (Loff 2014, p.2). This was, thus, the context of the eruption of popular anti-communist violence in the so-called *Hot Summer of 1975,* particularly in the north and central regions of Portugal, which were rural areas mainly composed of Roman Catholic small landowners. These landowners did not receive well the socialist proposals of the Revolution Council in power and, in some cases, responded to the call of reactionary individuals and organisations to show their dissatisfaction by attacking Communist Party headquarters (Cerezales 2003). Simultaneously, two armed organisations (the ELP and the MDLP) were steering and contributing to these popular activities from Spain, composed of conservative individuals who sought exile in this country after the unsuccessful coup of 11 March 1975 (Dâmaso 1997). These were organisations that in the midst of a clearly divided world characterised by the bipolar rivalries of the Cold War, and of a Portugal on the verge of a civil war,^11^ considered that they would be able “to invade the country and defeat the communists by force of arms” (Dâmaso 1997, p.11).

The denouement of this very troubled period in Portuguese political history came on 25 November 1975, when a coup attempted by the extreme-left sector of the military was frustrated by Coronel Ramalho Eanes’ troops, giving origin to an institutionalized democratic regime (Maxwell, 1999). Thus, 25 November 1975 marked the end of the armed offensive from the extreme-right (Dâmaso 1997), which saw its concerns regarding a potential communist invasion disappear. However, this date also laid the foundations for a new wave of political violence, this time conducted by the extreme-left (Costa 2004).

Thus, the FP-25 emerged in 1980 with two main purposes. First, to stop the actions of the extreme right once again in power due to the counter-revolution of 25 November 1975, which re-established the capitalist order, repressing the proliferous social movements and even some rights obtained by the workers after the April Revolution (Sousa 1992). Second, to attain a real socialist revolution (Costa 2004).

## State of the Art

In the Portuguese context, themes such as political violence and armed struggle have been very little explored, and the few existent studies have been mainly conducted through historiographic lenses and based on document analysis. This leaves a gap in the field of social and human sciences research, which takes into account, for instance, personal stories of involvement in politically motivated violence, as well as attempts to produce in-depth accounts of this social phenomenon. In this section, we will map the relevant literature about the Portuguese armed struggle which took place in the three different periods of time considered in this article.

In order to collect the sources mentioned, we performed an online literature search using a series of keywords. We also implemented the so-called “snowball method” to find additional possible relevant manuscripts by examining the reference lists of manuscripts that were considered suitable to include in the review. We decided to not focus on media coverage of the phenomena under study since this has been already done (see Soutelo 2015a).

### Pre-Revolution Period (1967-1974)

According to the Portuguese History Dictionary, armed struggle in Portugal took place through the republican activities conducted between 1927 and 1936, the assault on the military quarter of Beja in 1961 and the activities of the LUAR, the ARA and the BR (Serrão et al. 1999). This is corroborated by the definition proposed by *Estado Novo*’s History Dictionary, which describes armed struggle in Portugal as the “actions carried out by organised civil groups of a political-military nature and whose essential objective was the erosion of the rear guard of the regime, the realisation of initiatives whose nature and exemplary character could mobilize the population against the power of the *Estado Novo* or the preparation of an armed uprising” (Rosas and Brito 1996, p. 526). In this context, the LUAR, the ARA and the BR are equally perceived as the organisations which carried out armed struggle in Portugal. This is corroborated by Irene Pimentel’s History of the Opposition to the Dictatorship (1926-1974), which maps the different types of opposition suffered by *Estado Novo*’s regime since its conception, until its downfall, also including the armed struggle conducted by those three organisations (Pimentel 2014).

The doctoral research conducted by Miguel Cardina on the Portuguese Marxist-Leninist organisations, which resulted in a book (Cardina 2011), filled the knowledge gap about this type of organisations. However, it did not give a primordial place to politically motivated violence committed by such organisations. The same has happened with other studies focussed on the student protests against *Estado Novo*’s regime, the labour movement, the political repression or the opposition to the Colonial War. The only exceptions are the doctoral research of Ana Sofia Ferreira and Raquel da Silva which specifically studied the armed organisations which fought *Estado Novo*’s regime (Ferreira 2015a, b; Da Silva 2016).

The majority of the literature produced on the history of the Portuguese Communist Party (PCP) ignores the debates around the use of politically motivated violence which happened from the late 1950s, with the exception of João Madeira’s book. This author explored the PCP’s dynamics which caused the postponement of their involvement in the armed struggle until the 1970s (Madeira 2013).

In the 1980s, there were two important scholarly contributions regarding the rise of the Portuguese radical left and armed organisations made by Martins and Loureiro (1980a, b). Despite some errors and omissions, these were for quite some time the more in-depth studies on the subject of politically motivated violence against *Estado Novo*’s regime.

Rui Bebiano wrote about the social context lived in Portugal in the 1960s. This context was marked by accounts of international armed violence (e.g., Latin America, Vietnam, Palestine, Northern Ireland), of our own Colonial War, of the first movements of the exiled Portuguese opposition (e.g., the assault on the Portuguese ocean liner ‘Santa Maria’ in 1961) (Bebiano 2006a) and of the rise of armed struggle in Portugal.

There is also one article published by Tereza Viegas dedicated to the BR, listing the actions carried out by this organisation, as well as its ideological influences and aims (Viegas 1996).

A few former pre-revolution militants have also been prolific regarding the publication of their memoirs. Among them Camilo Mortágua, one of LUAR’s leaders (Mortágua 2013), Francisco Miguel (1977), Jaime Serra (1999) and Raimundo Narciso (2000), members of ARA’s central command and Carlos Antunes, one of BR’s founders (Antunes 1974). Some pre-revolution militants have opted for a semi-academic route, combining some research (mainly document analysis, but also interviews) and their own personal experience. Fernando Pereira Marques from LUAR, for instance, published his first book in 1976 where he gathered some of the documents he had written about the organisation (Marques 1976), and recently combined his personal testimony, the testimonies of other LUAR militants, abundant documentation and his experience as a historian to produce his latest book about the armed organisations which contributed to the fall of *Estado Novo*’s regime, in particular the LUAR (Marques 2016). Two more examples are the work of Hipólito dos Santos on the years he spent with LUAR (1967-1970), where he covers the activities of the organisation, its structure, financing and infiltrations (Santos 2011) and the work of Isabel do Carmo, one of BR’s co-founders, who recounts her experience of life underground, as well as other similar armed struggles across Europe in the 1960s and 1970s (Carmo 2017). Finally, the journalist Isabel Lindim, Isabel do Carmo’s daughter, has been collecting documents about the BR since 2007 (thereby establishing a physical, as well as a digital archive) and interviewed fourteen former BR women, which in turn led to her writing a book about their experiences (Lindim 2012). However, it is interesting to note that in some of the books (e.g., Narciso 2000; Lindim 2012) pseudonyms are still kept, because people fear discrimination in their social circles.

### Counter-Revolution Period (1975-1976)

Literature concerning the right-wing armed organisations which fought in the couple of years that followed the April Revolution is even scarcer. The first two books on the subject were authored by journalists, one Portuguese and the other German, and published in 1976. Carlos Dugos’ book is very interesting because it is solely based on interviews with both ELP and MDLP militants living in Spain and preparing a counter-revolution that would restore order and stop a perceived communist invasion in Portugal (Dugos 1976). Günter Wallraff’s book is developed from his own covert operation within MDLP. This journalist aimed to reveal this organisation’s plans with regard to a counter-revolutionary coup in Portugal (Wallraff 1976).

However, the most comprehensive work on this subject was produced by Eduardo Dâmaso. This book covers the involvement of the Catholic church in the counter-revolutionary activities of right-wing organisations, such as the MDLP or the Maria da Fonte Movement, discusses the counter-revolutionary activities of the ELP and covers the polemic Bomber Network, which in 1976 committed daily attacks in the country (Dâmaso 1997). In the same vein, the journalist Miguel Carvalho has recently published a book on the armed violence carried out by the ELP and the MDLP after the April Revolution, trying to demonstrate that this period was characterized by enormous violence (Carvalho 2017).

Riccardo Marchi has also produced a great amount of literature on the Portuguese right-wing, however, he only deals superficially with the political violence committed by organisations such as the ELP or the MDLP (Marchi 2012).

There are also two books of memoirs produced by MDLP militants. The first one was authored by Alpoim Calvão, MDLP’s operational leader. It covers his life from the moment he moved with his family to the Portuguese colony of Mozambique as a 14-year-old boy until his involvement in the creation of the MDLP after the April Revolution (Calvão 1976). The second one was authored by Rui Hortelão, Luís Sanches Baêna and Abel Melo e Sousa and is about Alpoim Calvão’s life. The authors consider it as “an almost biography” (Hortelão et al. 2012).

### Post-Revolution Period (1980-1987)

On the subject of the FP-25, two militants’ memoirs are recorded. One of them – The Ashes of a Lost Time – was published in 1985 by one of the repentants (i.e. a militant who after arrest decided to collaborate with the police) of the organisation. The book is, in some way, an attempt to justify his militancy in the FP-25, as well as its choice of denouncing the organisation to the police (Macedo 1985). In the other – Asphalt Guerrilla: the FP-25 and the Portuguese Time – the author tells of his experience as a militant in the FP-25 (Sousa 1992).

The FP-25 was also the focus of two other books. One written by a former inspector of the judiciary police – Terrorism and the FP-25 Years Later – which covers in detail the organisation’s activities (Costa 2004). The other written by a journalist – To Live and to Die in the Name of the FP-25 – which equally covers the organisation’s activities, but also the whole judicial process, including interviews with judges, magistrates and judiciary police inspectors (Vilela 2005).

## The Post-Dictatorship Memory Politics and Political Narratives in Portugal

The debates surrounding the Portuguese armed struggle have been historically undervalued by the social and human sciences in this country. This is a phenomenon that even takes place in the context of research projects on the opposition to *Estado Novo*’s regime or the revolutionary period of 1974-1975. Such research projects tend to be limited to a generic and superficial approach. Even militants’ memoirs only began to appear, with one or two exceptions, from the late 1990s onwards, becoming more common in the early twenty-first century.

This scenario leads to the question: why is this so? We argue that the answer to such a question starts in the late 1970s with the rise of post-dictatorship memory politics, which encompassed a historical revisionism that sought to whitewash the memory of the dictatorship and deny the revolutionary genesis of democracy in Portugal.

However, rewinding a few years, it is possible to see that it was not always so. In the two years between 25 April 1974 and the adoption of the constitution in April 1976, there was a “liberation of memory from oppression” (Loff 2015, p. 29), expressed through, for instance: the tributes paid to the victims of the political police and to the anti-fascist resistance, the publication of texts in which *Estado Novo*’s repression in Portugal and in the colonies was denounced, the publication of memoirs of the ex-prisoners of the Tarrafal concentration camp in Cape Vert and of the anarchists of the 1920s and 1930s who fought the regime. Although at this point, the anti-fascist memory gained a clear hegemony in the political narratives, it did not last long. In fact, the 25 April 1974 Revolution and the Ongoing Revolutionary Process^12^ which followed it, quenched the memory of the organisations that carried out armed actions against *Estado Novo*’s regime, because the opposition’s preferred political narrative did not focus on such acts nor did it value them. Actually, such organisations, as well as their armed activity, started to be perceived as marginal and, to some extent, eccentric, a sort of exception in the history of resistance, which did not deserve to be framed academically. To this contributed the circumstances that started in the morning of the 25 April 1974. The captains’ military coup became a revolution that gave origin to moments of historical acceleration, as was the case of 28 September 1974^13^ or 11 March 1975.^14^ In this context, it was mainly the radical left groups, whether they had carried out armed actions against the dictatorship or had only theorized about this necessity, who worked towards the radicalisation of the process. Their objective was the seizure of power by the workers and the creation of a socialist society. From March 1975 onwards, these sectors of the radical left were the protagonists of some of the most emblematic confrontations of the Ongoing Revolutionary Process: the occupation of the newspaper *República* and of the *Radio Renascença*, robbing them both of the tutelage of the Socialist Party (the main centre-left party in Portugal) and of the Catholic Church; as well as the Spanish embassy’s assault, which happened as a radical form of protest for the death sentence of three FRAP (Anti-Fascist and Patriotic Revolutionary Front) and two ETA (Euskadi Ta Askatasuna) militants, which were two of the main anti-Franco organisations (Rosas 2004). In addition, the most reactionary sectors of the Portuguese right-wing also decided, in the Summer of 1975, to take part in armed violence. These organisations were based in Spain and in the north of Portugal and had the support of sectors of the Catholic hierarchy and right-wing parties (Dugos 1976). Both organisations urged northern populations to attack and set fire to left-wing parties’ headquarters (Cerezales 2003). In this period, extreme-right organisations were also part of larger operations, such as the murder of Father Max in a bomb attack, and the bomb explosion at the Cuban Embassy in Lisbon, which killed two embassy employees. These were just two among dozens of other bomb attacks against people connected to the PCP and other leftist organisations (Dâmaso 1997; Carvalho 2017).

The military movements or counter-revolution, as it is called by some, of 25 November 1975 closed the revolutionary phase of the process of transition to democracy, which meant the defeat of radical leftist organisations and movements in Portugal (Cerqueira 2015). Such a defeat was further legitimised by the loss of the left in the constitutional elections of 1975, as well as in the presidential elections of 1976. From then on, a new stage began with the institutionalization of a representative democracy. In this context, the triumphant political sectors sought to halt and defuse the revolutionary transformations that had occurred in the previous two years, even though they were constitutionally consecrated (e.g., the agrarian reform and various nationalisations) (Rosas 2004). This new framework, the herald of political stability, allowed Portugal to formally submit an application to the European Economic Community (EEC) in March 1977. This process of institutionalization of democracy in Portugal was also accompanied by an effort to reconcile Portuguese society, creating an environment that allowed the absence of condemnation and an amnesty for those, both from the political right and left wings, who had engaged in armed violence (Camacho 2011). These aspects were, then, the foundation for the reconfiguration of memorialist discourses and of memory politics, creating political narratives which deliberately devalued the memory of the anti-fascist resistance and of the April Revolution, and which rewrote the more recent history in an objective process of historical revisionism (Rosas 2016).

Thus, in Portugal, from the late 1970s to the early 1990s, there was a clear devaluation of the memory of resistance to the dictatorship, both regarding its peaceful and violent components. This coincided with the country being governed by the right-wing from 1978 until 1995. These years were marked by a serious economic and social crisis, which was blamed, by the political and economic elites, on the revolutionary process. In their view, such a process was responsible for the country’s economic problems due to the political confrontations it caused, to the decolonisation and to the sectarianism of the parties whose political project had been defeated on 25 November 1975 (the PCP and the radical left). Moreover, the political project of Cavaco Silva, prime minister of Portugal between 1985 and 1995, neoconservative and economically neoliberal, required the rejection of the Portuguese Revolution as a historical experience. He stressed that “the Portuguese society lived in 1974-1975 a period of exacerbated political agitation and of predominance of ideological discourse” (Silva 1987, p. 386), thus contributing to the demonization of the Ongoing Revolutionary Process. At this point, such a process came to be seen as responsible for almost causing a civil war and tended to be described with adjectives in the semantic field of *madness* and *disease* (Loff 2015, p. 65). However, it also served to underline the fact that it was a process that only the moderate forces which led the 25 November 1975 managed to stop. Furthermore, from 1989 onwards, the Portuguese government adopted a totalitarian reading of the April Revolution, arguing that the leftist forces aimed to replace *Estado Novo*’s regime by a communist/Marxist dictatorship (Loff 2015, p. 73). All these efforts, once again, implied a negative memory of the April Revolution and of the revolutionary process. In this vein, the rhetoric of the pacification of the Portuguese society and of the reconciliation with the past blocked and inhibited any debate on revolutionary violence, anti-fascist resistance, *Estado Novo* or April Revolution.

Despite having taken on such specificities in Portugal, the historical revisionism phenomenon is in some way underpinned by its emergence in most Western European countries, accompanied by the political rise of right-wing parties, as well as neoliberal and neoconservative political, economic and social values throughout the 1970s and 1980s, in the context of the crisis of Marxism and of the Left. However, it became a social phenomenon in the 1990s, coinciding with the deepest crisis of the left which followed the implosion of the Soviet bloc (Soutelo 2015b, p. 216). In this context, Fernando Rosas considers that the legitimising ideological paradigm of most post-war Western societies undergoes an unprecedented subversive pressure with the advent of Thatcherism and of Reaganism and the overthrow of the Soviet world, designed to legitimize the establishment of a new neoconservative and capitalist course (Rosas 2016, p. 62). Additionally, Pier Paolo Poggio argues that in the post-1989 world, anti-communism was the link among the various historical revisionisms and the liberal and conservative political forces it represents (Poggio 2006, p.211).

Manuela Cruzeiro identifies two main currents of historical revisionism in the Portuguese historiography: the first one is focussed on continuity, by devaluing ​​the April Revolution and its consequences and emphasising a continuity between dictatorship and democracy; and the second one, supported by most people, writes the history of the victors, analysing the history of the April Revolution centred on their perspective (Cruzeiro 2011, pp. 126-131). The first current perceives the April Revolution as a historical breakdown, objectively whitewashing the previous regime. The second current, politically steeped in the triumphant regime brought by the 25 November 1975 counter-revolution, privileges the historical reading which recognises the role of the winning political and military forces. It underestimates the role of other readings which seek to highlight the role of social movements and the role of political violence in the transition process. This is an elitist, partial historical reading that implies the implicit or explicit condemnation of the political organisations of the radical left. In this sense, the reading of the political violence which took place during the dictatorship, in the April Revolution and as a consequence of it, is done from a negative and prejudiced perspective.

The historical revisionism which marked the Portuguese society in the twenty years after the April Revolution was also strongly supported by the fact that the main archives for the study of the dictatorship were kept closed for a very long time (until 1996). This documental obstruction was justified in public discourses by those responsible for the archival politics in the country (among them Borges de Macedo, director of the National Archives between 1990 and 1996) as an attitude of caution, particularly with regard to military archives. According to such views, it was necessary to exercise extreme caution when accessing contemporary sources, since they could contain personal information regarding numerous figures of Portuguese political and social life, who could be still alive or who could be dead and whose memory would be tarnished (Rocha 1990; Braga 1994). This was, in essence, a policy that, according to Fernando Rosas cited in Loff (2015, p. 89), was “marked by strong hostility giving access to researchers”.

For the history of the organisations which embarked on armed struggle, the issue of access to archives by researchers still poses another problem: the lack of systematised archives per organisation, circumscribing the documentary territory to the archives kept by the police, the judicial system and individuals. Actually, the majority of the information regarding pre-revolution organisations can be found in the political police archive. Obviously, such documents cannot be ignored, but they equally pose serious difficulties to the researcher due to their nature. Firstly, most information was obtained coercively and with the use of flagrant violence. Secondly, the police interrogation records are composed of inaccurate, often lacunar, and very partial questions and answers. Thirdly, in their reports, the police commonly omitted or falsified facts or objects of the investigation in order to extract evidence that could support a conviction in court and provide data that could be investigated by other individuals (Pimentel 2007; Cardina 2010). Therefore, although this archive is a fundamental source of documentation, it requires more robust questioning and should not be used as standalone information. In terms of the documentation regarding both counter- and post-revolution organisations, it is mostly dispersed throughout the different courts where the trials were conducted. In addition, on the one hand, such courts do not tend to organise their files, which creates difficulties in finding them, and, on the other hand, researchers often simply see their access requests denied. Finally, a considerable amount of documentation lives in personal archives, access to which depends on the empathy or trusting relationship that can be established with the researcher. However, in recent years, there has been a change of attitude towards personal archives, with many of their owners depositing them in archival institutions such as the 25 April Documentation Centre or the Mário Soares Foundation. This allows the preservation of the historical memory and provides the possibility of making them accessible to research.

Nevertheless, according to Manuel Loff, from the 1990s onwards we have been experiencing a “rebellion of memory” (Loff 2000, pp. 189-199), as a reaction to twenty years of aggressive historical revisionism which devalued the memory of anti-fascist resistance, particularly regarding the armed organisations. Consequently, in this period, public debates concerned with a “sheer whitewashing of the dictatorship” by right-wing governments began to take place (Loff 2014, p.10). Such debates were triggered by the public acknowledgement that former political police members were receiving pensions for “exceptional and relevant services rendered to the country”, as well as retired military men who fought in the Colonial War, while Captains of the Revolution, such as Salgueiro Maia, were denied their pensions. These were circumstances that showed a clear depreciation of the military men who contributed to the overthrow of the dictatorship. However, the last straw, according to Loff (2014), came in 1994 when a commercial TV station organised a debate between a former political police member (Óscar Cardoso), a historian who had been a political prisoner (José Manuel Tengarrinha) and a former member of the Revolution Council (Sousa e Castro), giving the opportunity to the first to draw a positive picture of the political police and to deny the torture practices and executions carried out by this institution during the dictatorship. This debate raised serious controversies, leading, for instance, the republic president at the time and anti-Salazarist resistant Mário Soares (1994) to affirm: “it was as if [Klaus] Barbie – the butcher of Marseille – had been invited to a debate with his own victims. […] What are we going to tell our children after they have watched this debate on TV? That we have imagined this whole story? What on earth will be our legacy?” A few right-wing personalities equally contributed to this debate, reiterating their perspective that the April Revolution was undoubtedly worse than Salazar’s dictatorship, and recalling the atrocities that in their opinion happened in the country afterwards – “nobody recalled the arbitrary arrests, censorship, intimidation, tipping off, manipulation, slander, death threats, torture and injuries, squatting in properties, purges, destruction and other post-April 25^th^ savagery. It was not the PIDE [political police] who did those. It was the MFA-PCP [Movement of the Armed Forces – Portuguese Communist Party] alliance of rejuvenating memory, that true spittle on our country’s democratisation process” – and in the colonies as a result of the decolonisation process – “they [the leftist individuals composing the Revolution Council] preferred heroically, to abandon to their fate [in Angola and Mozambique] millions and millions of people who, in the meantime, died in one of the most terrifying catastrophes in the history of mankind and whose responsibility is all theirs” (Moura 1994).

Moreover, the start of this “rebellion of memory” also coincided with the beginning of a new political cycle marked by the coming to power of the Socialist Party. For the first time, on its 25th anniversary, the April Revolution was celebrated with enthusiasm by the political establishment. At this point, a stimulus was provided for the publication of autobiographical accounts and memoirs by some of the protagonists of the resistance to the dictatorship. In their old age, they became aware of the need to fix the autobiographical narrative, since they saw themselves confronted with the proliferation of revisionist discourses, which denied the repressive past. Among these publications are the ones cited above which were authored by pre-revolution militants. As it is possible to see by the number of publications, LUAR’s militants were the most concerned about telling their stories. This might be due to the fact that the LUAR was an organisation with very specific characteristics, distanced from the more traditional forms of party logic and structure. For instance, its one and only ideological stance was being anti-fascist. In addition, it was the only organisation that did not commit bomb attacks and which always kept its operational base outside the country, both in Paris and Brussels. However, despite being strongly committed to telling the story of the organisation until the April Revolution, LUAR’s militants did not cover its story during the Ongoing Revolutionary Process. This was a period of time in which the LUAR developed a remarkable work in conjunction with other social movements (Ferreira 2015a). Thus, the LUAR’s story after the April Revolution still needs to be told. As for the ARA, the PCP’s strategy during the period of historical revisionism was to avoid talking about issues that could raise controversy and incite criticism by the right to the role of the Communist Party in the history of anti-fascism and the April Revolution. Moreover, it was not until the end of the 1990s when the concept that devalued individual militancy experiences to the detriment of collective effort began to be reconfigured, which ultimately led to a silencing of the memorial discourse of many resistance militants (Nogueira 2009). Regarding the BR, there is only one very recent half memorialist, half academic piece. This can be explained by the intense revolutionary activity developed by the Revolutionary Party of the Proletariat (PRP), to which the BR was coupled, in the period after the April Revolution. Despite the suspension of armed struggle right after the April Revolution, the BR were reactivated and re-established in August 1975, returning to the commission of armed activities, such as bank robberies, in order to finance the organisation. Such activities led to the imprisonment of BR’s co-founders and leaders Carlos Antunes and Isabel do Carmo, in June 1978.

The right-wing return to power in 2002 influenced the celebrations of the 30th anniversary of the April Revolution since again such governmental elite was bothered by the revolutionary legacy. The slogan of these celebrations – April is *Evolution* – instead of *Revolution*, provoked a strong reaction in the media and in the public space. The right-wing strategy of imparting an evolutionary character to the April Revolution, in tune with the idea of transition, was completely torn by the political reaction in defence of the April Revolution as a specifically Portuguese model of building democracy (Loff 2015). Such a socio-political context was favourable to the dismantling of the establishment’s discourse since the government was quite unpopular. This could be seen by the heavy defeat suffered by the right-wing two months later in the European elections of that year, as well as, in the following year, 2005, the fact that the right-wing experienced the worst result in the legislative elections of the last 30 years.

During the difficult years of the recent economic crisis (2011-2015), which mainly affected the middle and low classes, at a time of lack of hope, there was a memory recovery regarding the more radical forms of struggle. In the face of the apparent ineffectiveness of peaceful demonstrations, the deaf ears of politicians, the discourse of inevitability, and the sense of powerlessness to change the circumstances, there was once again a political narrative linked to the possible need for a violent response to reverse the situation. However, the recalled violence focused mainly on the pre-revolution period, in which the struggle against the dictatorship and the Colonial War, and the fact that blood crimes were not committed, seem to justify a certain kindness towards political violence. In this sense, the armed activities committed in the whole period of the counter- and post-revolution ended up falling into oblivion. The country went through a democratisation process and the existence of political violence in democracy became the unspoken facet of the armed struggle history in Portugal.

In this context, there is a clear gap in the scholarly knowledge regarding the Portuguese armed struggle, except for Miguel Cardina’s work on Maoism in Portugal between 1964 and 1974 (Cardina 2010), Ana Sofia Ferreira’s on armed struggle organisations during the dictatorship (Ferreira 2015a) and Raquel da Silva’s on the narratives of political violence in Portugal both before and after the April Revolution (Da Silva 2016).

## Conclusion

The purpose of this article was to explore the reasons behind the low number of both lay and academic publications regarding the Portuguese history of armed struggle, both before and after the 25 April 1974 Revolution. We argued that the main reasons are the whitewashing of *Estado Novo*’s regime and the consequent historical revisionism which took root in the country in the late 1970s and throughout the1980s. In this sense, we demonstrated how part of the Portuguese historical identity has been manipulated by political and ideological interests and motivations, which influence the collective memory. However, we also demonstrated how memory has become, from the 1990’s onwards, a culturally and politically intense battle field in Portugal, in which the different sides have made efforts to tell their stories and to influence the nation’s political narratives, as well as what should be remembered and what should be forgotten.

Nonetheless, Portuguese public opinion at present is still fairly influenced by revisionist perspectives. Consequently, the stories of those individuals who invested a significant part of their lives fighting violently for their political ideals have still been predominantly ignored and erased from the collective memory. There is, for instance, a clear lack of acknowledgement of their existence (and of political armed struggle in general) in the history books and school curriculums in Portugal. This is something that the Portuguese historian Rui Bebiano (2006b, p.9) calls “unmemory” rather than “forgetfulness”, because while the latter involves “carelessness, accident, casual blur of past reminiscences”, the former involves “a voluntary erasure of memory, a lack of knowledge or even a lack of interest in certain areas of living, considered irrelevant and not instrumental”.

Therefore, it is the responsibility of researchers in the social and human sciences to rescue the study of political violence in Portugal and to bring it to the collective memory arena.

## Footnotes

1 We acknowledge that some of the matters approached here are also relevant in other dictatorial contexts (e.g., Spain under Franco, Italy under Mussolini, Greece under the Cornels). However, it is not our ambition to explore them in this article, due to the limited space at our disposal.

2 *Estado Novo* (New State) is the designation given by António de Oliveira Salazar to the regime he gave birth to and intended to rule. *Estado Novo* represented the embodiment of power and a strong state organized around a strong figure – Salazar was the answer to the claims of the reactionary factions of the bourgeoisie, of a good part of the Catholic Church, and of some intellectuals during the First Republic for a strong and competent government, which could bring an end to the existing financial, political, social and cultural turmoil (Amaro 1982).

3 The revolution of 5th October 1910 marks the end of the Constitutional Monarchy in Portugal (1820-1910) and the beginning of the First Republic. This political period was always followed by great difficulties of consolidation, which were evident through the existence of a deep political, economic and social crisis, manifested, for instance, in the existence of successive governments, in the assassination of the republic president Sidónio Pais, in 1918, and in the ten coup attempts between 1921 and 1926 (Mattoso 1994; Saraiva, 1993).

4 Salazar was very critical of the financial policies put in place by the July 9th government headed by Óscar Carmona in the presidency, and by Sinel de Cordes in the Finance Ministry, which took shape through various newspaper publications, where he started building his image as a very serious and competent man (Pinto 2010).

5 The first time was in the first year of the military dictatorship and only lasted a few days (3rd – 19th July 1926), because the government he was part of was quickly replaced.

6 This exclusion is related to the fact that a fascist dynamic, where a single party mobilises the masses and seizes power, was not at the origin of *Estado Novo*. In reality, *Estado Novo* was originated by one individual who took control of the government and transformed a military dictatorship into a dictatorial regime (Nolte, 1966).

7 Democratic and liberal republican opposition which from the start opposed the dictatorship established in Portugal on 28th May 1926, and tried several times to restore the regime initiated on 5th October 1910.

8 The unforeseen defeat of General Humberto Delgado in the electoral process of 1958 was not accepted as free or credible, causing the opposition to start questioning the heretofore defended reformist measures.

9 Actions originated both from priests and lay groups and essentially marked by the arrest of the priest Mário de Oliveira, who used to preach against the Colonial War; and by the *Capela do Rato Watch,* a initiative that happened on the 1972-1973 new year’s eve and stated the Catholic church position against the Colonial War and the dictatorial regime.

10 In January 1974 thirty students were arrested.

11 The Revolution caused a partisan run for power among the left forces repressed during the dictatorship (e.g. Communist Party) and some emergent ones (e.g. Socialist Party) and an inexistent right that was trying to organize itself around figures of the former regime. This partisan game ended by dividing society into ‘good and bad guys’, or ‘democrats’ and ‘reactionaries’ respectively, giving birth to deep hatred, injustices and violence committed by both sides (Dâmaso 1997, p.14).

12 Commonly known in Portuguese as PREC – *Processo Revolucionário em Curso*.

13 This was the day of a mass demonstration planned by General António Spínola, the then President of the Republic, in order to show that the majority of the population did not support the radical ways in which the MFA (Armed Forces Movement) was running the country. However, the demonstration was blocked by the MFA and the president sent into exile in Spain.

14 On this date, there was a failed attempt at an anti-communist coup led by General António Spínola, which contributed to the further radicalisation of the Ongoing Revolutionary Process, including arrests of figures of the fallen regime.
